# Characterization of genomic variants associated with congenital heart disease in patients from southwestern Colombian

**DOI:** 10.1016/j.heliyon.2023.e23678

**Published:** 2023-12-14

**Authors:** Angie Lizeth Grueso Cerón, Daniela Arturo-Terranova, José María Satizábal Soto

**Affiliations:** a Universidad del Valle, Faculty of Natural and Exact Sciences, Department of Biology, Biology Program, Cali, Valle del Cauca, Colombia; b Universidad del Valle, Faculty of Health, School of Basic Sciences, Postgraduate in Biomedical Sciences, Cali, Valle del Cauca, Colombia

**Keywords:** Congenital heart disease, Genes, *In silico* models, Exome sequencing

## Abstract

Congenital heart diseases correspond to errors during embryogenesis, generating structural and functional malformations. Congenital heart diseases are the most prevalent congenital malformations and are responsible for the highest infant morbidity and mortality. Among these cases, 8 % can be attributed to variants in genes associated with cardiac development. To establish the population frequency of genomic variants that cause congenital heart disease, a review of the scope of prevalent genes was carried out, complete exome sequencing results of 320 patients without suspicion of congenital heart disease were used, the exome sequencing is a technique based on DNA extraction using a Qiagen kit, with massive sequencing of Nextera TM libraries using an Illumina platform with 100X coverage, alignment with reference genome GRCh38/hg19, and analysis with the CRAVAT program; clinical characterization, significance classification, and gene interaction networks were performed. The scope analysis allowed to determine that the genes *NKX2-5, TBX20, GATA4, NOTCH1, PTPN11* are the most prevalent, the variants with the highest allelic frequency were c.63A > G (0.2844), c.39T > C (0.3406), c.1132A > G (0.0406), c.1669+9T > C (0.3531) and c.854-30T > C (0.0875) respectively; 4 variants were reclassified by in silico tools, in the *NKX2-5* gene c.335-311_335-303del from benign to probably pathogenic, in the NOTCH1 gene c.2354-24C > T from benign to pathogenic, and in the *PTPN11* gene c.2354-24C > T and c.854-30T > C from benign to pathogenic. 17 % of intronic variants and 4.8 % of missense variants were identified. This work contributes to knowledge about the frequency with which genomic variants associated with congenital heart disease are present in the population, generating a tool for early diagnosis, early treatment, thus reducing morbidity and mortality, with a view to future universal molecular neonatal screening of congenital heart disease.

## 1 Introduction

The process of cardiac formation, carried out during the first trimester of pregnancy, is a complex process that includes proliferation, differentiation, cell migration, septation, chamber formation, formation of the cardiac conduction system, these processes are controlled by transcription factors, cofactors, cell signaling molecules, among others [1,2]. Any genomics variants that compromise some of these genes could cause the so-called congenital heart disease (CHD) [3]. CHD is defined as functional structural malformation of the heart or great vessels, present at birth, due to errors during embryogenesis [4]. 8 % of CHD are due to variants in genes that regulate cardiac development [5]. Considered one of the most prevalent malformations and the cause of the highest infant morbidity and mortality, it affects approximately 1 % of births per year, with a global prevalence of 0.5–9 per 1000 live births (LB) [[6], [7], [8]] and a prevalence in Colombia of 2.4 per 1000 births. In Colombia the National Institute of Health (NIH), includes CHD within malformations of the circulatory system, where they occupy the first place of prevalence with 39.8 per 10,000 LB [9,10].

Advances in genomic sequencing and bioinformatics technologies have allowed identify CHD associated gene variants and perform rapid data analysis genetics, which contributes to the recognition of gene sequences, analysis of gene expression, organization, and relationship of biological information, helping the processing and elucidation of the molecular etiology of these pathologies [11,12].

The recognition of the population frequency of genomic variants that may be associated with CHD, making use of bioinformatics techniques and *in-silico* analysis will allow progress in timely diagnosis, transdisciplinary specific management and a decrease in morbidity and mortality.

## 2 Methods

A cross-sectional, descriptive, non-experimental study was carried out, scope review, genomic characterization of the variants, calculation of allelic frequency and gene interaction networks. The scope review was carried out using the databases: Pubmed, Scopus and Lilacs of articles published between 2010 and 2023, in English and Spanish, of the genes commonly related to CHD.

The search equation used was the following:((gene) AND (congenital heart disease)) AND (prevalence)

**Inclusion criteria used**: In order to identify possible variants associated with CHD in the general population, patient with complex disease, without clinical suspicion of congenital heart disease with complete exome sequencing (WES) results obtained from the database of the Institute of Medical Genetics - GENOMICS (Cali-Colombia).

**Exclusion criteria used**: Patients with a confirmed molecular diagnosis of congenital heart disease.

For the study data, the results of WES of 320 patients without suspicion of CHD, and with complex disease, belonging to the database of the Institute of Medical Genetics - GENOMICS (Cali-Colombia) were used, using the VCF Editor. 1.0 created in the doctoral thesis "Representation of the Genomic Variability of the Mucopolysaccharidosis Complex in the Southwest Colombian" [13], which allowed editing the VCF format of the exomes, and converting it into Excel format, with the aim of applying the different search filters and obtaining a final file with the information of interest, displaying columns organized according to chromosome, position, altered amino acid and record in Clinvar; the exome sequencing is a technique based on DNA extraction using a Qiagen kit; Subsequently, massive sequencing of Nextera TM libraries was performed using the Illumina platform with 100X coverage. An alignment was executed with the reference genome GRCh38/hg19, subsequently analyzed with the CRAVAT program that provides high-throughput services for researchers to annotate and prioritize genes and exome variants on a small scale. Annotations at the level of variants, genes and bioinformatic scores are provided to allow interpretation and prioritization of variants identified in sequencing studies, including frameshifts, insertions/deletions, splice site, missense and nonsense, performing a variant mapping (genome transcripts <−> protein sequence <−> protein structure <−>), allowing millions of variants to be evaluated in a single step; It is useful to facilitate high-throughput screening, and provide predictive scores for germline variants.

The variants found in the genes associated with CHD in the database were analyzed by means of a search in population, genome, disease, and sequence databases to verify whether they had been previously described. The pathological implications were determined through bioinformatics tools, using *in-silico* technology that allows predicting the consequences of genetic variants on protein structure and function.

**Population databases**.

− 1000 Genomes Project (https://www.internationalgenome.org/): It presents free access on the web and allows a friendly search for the variants reported so far in the population.
- Exome Aggregation Consortium (Exac) (http://exac.broadinstitute.org/): Platform that seeks to aggregate and organize exome sequencing data from a variety of large-scale sequencing projects, and make summary data available to the broader scientific community. The data on this website covers 60,706 unrelated individuals sequenced as part of several population- and disease-specific genetic studies.
- Genome Aggregation Database (gnomAD) (https://gnomad.broadinstitute.org/): It is a resource developed by an international coalition of researchers, with the goal of aggregating and harmonizing genome and exome sequencing data from a wide variety of large-scale sequencing projects, and making the summarized data available to a broader audience.

Genome databases.

- Ensembl Genome Browser (http://www.ensembl.org/index.html): The project began in 1999 with the ultimate goal of automatically annotating the genome, integrating said annotation with other available biological data, and publishing all this information online and free of charge. All annotations are integrated with a huge number of external reference sources, making Ensembl a unique integrative resource.

Disease databases.

- Online Mendelian Inheritance in Man (OMIM) (https://www.omim.org/): It is a complete and authoritative compendium of human genes and genetic phenotypes that is available and updated daily.
- Clinvar (https://www.ncbi.nlm.nih.gov/clinvar/): ClinVar is a freely accessible public archive of reports of the relationships between human variations and phenotypes; facilitates access and communication about claimed relationships between human variation and observed health status, processes submissions reporting variants found in patient samples, claims made regarding their clinical significance, submitter information, and other data back.
- Varsome (https://varsome.com/): Varsome is a search engine for human genomic variations that includes information from 30 databases in one central location.

### 2.1 Sequence databases

-National Center for Biotechnology Information (NCBI) (http://www.ncbi.nlm.nih.gov/): The NCBI is the American public platform that serves as a global reference in biomedical and genomic information. By searching dbSNP, information is obtained on the possible pathophysiological effect of the variant and its frequency in the European population.

### 2.2 In silico methods

- Polymorphism Phenotyping v2 (Polyphen-2) (http://genetics.bwh.harvard.edu/pph/): Polyphen-2 predicts the impact that an amino acid substitution can have on the structure and/or function of a human protein through comparative considerations.
- UMD PREDICTOR (http://umd-redictor.eu/analysis.php): UMD predictor is an independent platform that contains all predictions for all substitutions of any human transcript. This tool provides a combinatorial approach to identify possible pathogenic variations, which associates the following data: localization within the protein, conservation, biochemical properties of wild-type and mutant residues, and the potential impact of the variation on the mRNA.
- SIFT (Sorting Intolerant From Tolerant) algorithm (http://sift-dna.org): SIFT classifies amino acid substitutions in a given protein and predicts whether these changes will cause a phenotypic effect on the protein. SIFT is based on the premise that the important amino acids of a protein are conserved in evolution, so changes in them must affect the functionality of the protein.
- Human Splicing Finder (HSF) (http://umd.be/Redirect.html): It is a tool to predict the effects of mutations on splicing signals or to identify splicing motifs in any human sequence. It has new position weight matrices to evaluate the strength of 5′ and 3′ splice sites and branch points.
- CADD.- Combined Annotation Dependent Depletion (https://cadd.gs.washington.edu/): It is a tool to score the deleteriousness of single nucleotide variants as well as insertion/deletion variants in the human genome. C scores strongly correlate with allelic diversity, pathogenicity of coding and non-coding variants, and experimentally measured regulatory effects.

### 2.3 Variant classification according to the American College of Medical Genetics (ACMG) was used [14]

- **Pathogenic:** variants that have strong evidence of association with disease.
- **Probably Pathogenic**: variants probably involved in disease since the nucleotide change presents a probability of more than 90 % of being pathogenic.
- **Uncertain significance (VOUS):** variants with possible functional changes, but with contradictory or insufficient evidence. That is, it cannot be defined if it is pathogenic or not.
- **Probably benign:** variants with evidence suggesting benignity, but with weak data in the literature that do not rule out biological and possibly clinical impact. The nucleotide change has a greater than 90 % probability of not being pathogenic.
- **Benign:** genetic variants that do not alter functionality.

The allelic frequency (AF) of each of the gene variants was calculated by simple count:

Allelic frequency = (Number of copies of the allele in the population)/(Total number of copies of the gene in the population)

## 3 Results

From the scope review, it was found that the genes prevalently associated with CHD are:

*NKX2-5:* It is located on chromosome 5q35.1, consists of 2 exons, and codes for the *NKX2-5* protein, a 324 amino acid transcription factor [15]. It plays an important role in regulating proliferation, differentiation and electrophysiological properties of cardiac cells (15). Variants in this gene are associated with septal defects, cardiomyopathy, outflow tract defects, left ventricular hypoplasia, and cardiac conduction system defects [16].

*TBX20:* It is located on chromosome 7p14.2, consists of 8 exons and codes for the *TBX20* protein considered a transcription factor [17]. It is one of the first transcription factors that are expressed in cardiac development, in the first and second cardiogenic field, endocardium, myocardium, endocardial cushions, heart valves and atrioventricular septum, acting mainly in the proliferation of cardiomyocytes (17). Variants in this gene are associated with defects in septation, valvulogenesis and cardiomyopathies [18].

*GATA4*: It is located on chromosome 8p23.1, contains 7 exons and encodes the *GATA4* protein, a member of the zinc finger transcription factors [3]. This gene is expressed in cardiac tissue, functioning as a critical regulator of cardiac differentiation, it acts in processes of proliferation, cell specification and differentiation, and cardiac morphogenesis, it is present in the formation of the cardiac tube, separation and development of the outflow tract, formation of the atrioventricular canal and development of the cardiac conduction system [3]**.** Variants in this gene are associated with septal defects, tetralogy of Fallot, atrial fibrillation, pulmonary stenosis [19].

*NOTCH1:* It is located on chromosome 9q34.3, contains 34 exons and codes for the NOTCH1 protein, a member of the *NOTCH* receptor family [20]. NOTCH receptors are expressed in early stages of development, related to the development and proliferation of cardiac cells [20]. Variants in this gene are associated with calcified aortic valve disease, bicuspid aortic valve (BAV), and tetralogy of Fallot [21].

*PTPN11*: It is located on chromosome 12q24.13, contains 16 exons and codes for the protein tyrosine phosphatase SHP2, a member of the PTP family. This gene is related to differentiation of cardiac progenitor cells, development of valves and endocardial cushions, maturation and separation of the cardiac cavity. This gene is primarily associated with Noonan syndrome, characterized by CHD including pulmonary aortic stenosis and septal defects [22].

**Figure 1:**
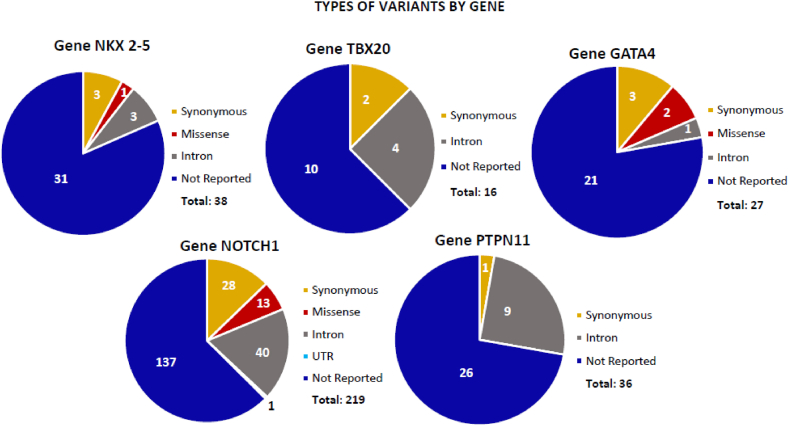
Number of variants and type of change of the NKX2-5, TBX20, GATA4, NOTCH1 and PTPN11 genes. A total of 336 variants were obtained, the NOTCH1 gene presented the highest number of variants.

In the results of the 320 WES analyzed, a total of 336 variants were found:

In the *NKX2-5* gene, 38 variants, 7 reported in disease databases: 3 synonymous, 3 intronics and 1 missense (Figure 1). By means of in silico tools, 6 variants were classified as benign and 1 (the variant c.335-311_335-303del (rs200467566)) was reclassified from benign to probably pathogenic (figure b; Table 1).

**Figure 2:**
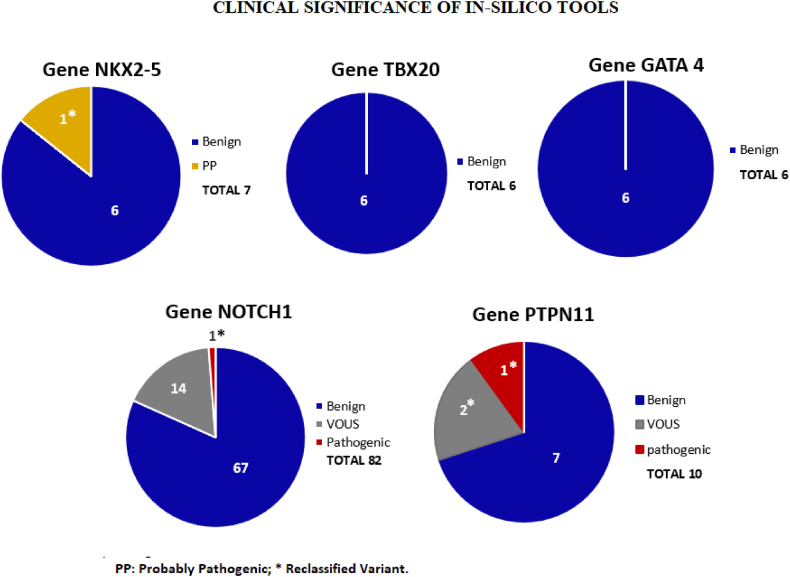
Clinical significance according to ACMG, generated by In-silico tools for the genomic variants of the genes NKX2-5, TBX20, GATA4, NOTCH1 and PTPN1, two variants were reclassified as pathogenic, one variant as probably pathogenic, and sixteen variants to vous.

**Table 1:** Variants reclassified by In Silico tools of the *NKX2-5, NOTCH1* and *PTPTN11* genes. Three variants were reclassified from benign to probably pathogenic and pathogenic.

Gene	Variants	Classification	Reclassification by In Silico tools
**NKX2-5**	c.335-311_335-303del	Benign	Probably Pathogenic
**NOTCH1**	c.2354-24C > T	Benign	Pathogenic
**PTPN11**	c.854-30T > C	Benign	Pathogenic

The three variants with the highest AF were c.63A > G (rs2277923) (0.2844), c.335-311A > T (rs3095871) (0.2688) and c.335-315A > T (rs55880439) (0.2500), values close to the population databases (Table 2).

**Table 2:** The three variants with the highest allele frequencies per gene and their comparison with population databases are shown.

GENE	Variant	Classification	AF in the present study	AF in 1000 genomes	AF in EXAC	AF in GnomAD
**NKX 2**–**5**	c.63A > G	Benign	0,2844	0,5357	0,4052	0,4323
	c.335-311A > T	Benign	0,2688	0,6110	0,4675	0,3964
	c.335-315A > T	Benign	0,2500	0,4030	0,4438	0,3933
**TBX20**	c.39T > C	Benign	0,3406	0,6939	0,6767	0,6792
	c.545 + 13A > G	Benign	0,2563	0,3440	0,3808	0,3890
	c.655-44G > A	Benign	0,2563	0,3440	0,3811	0,3890
	c.381-40A > G	Benign	0,0125	0,0030	0,0008	0,0010
**GATA 4**	c.1132A > G	Benign	0,0406	0,0430	0,0962	0,0952
	c.1000 + 23A > T	Benign	0,0281	0,0230	0,0066	0,0220
	c.1059C > T	Benign	0,0219	0,0390	0.01123	0,0100
**NOTCH1**	c.1669+9T > C	Benign	0,3531	0,8840	0,8704	0,8720
	c.2970-31A > G	Benign	0,3344	0,7650	0,6627	0,6380
	c.5473-43T > C	Benign	0,3281	0,7420	0,5920	0,5930
**PTPN11**	c.854-30T > C	Benign	0,0875	0,0180	0,0120	0,0180
	c.756 + 1274G > A	Benign	0,0781	0,1670	0,2108	0,1310
	c.854-21C > T	Benign	0,0281	0,0370	0,0592	0,0580
AF: Allele Frequency						

In the *TBX20* gene, 16 variants, 6 reported in disease databases: 2 synonymous, 4 intronic (figure a). By means of in silico tools the 6 variants were classified as benign (Figure 2; Table 1).

The three variants with the highest AF were c.39T > C (rs336283) (0.3406), c.545 + 13A > G (rs17675148) and c.655-44G > A (rs2072434) (0.2563), and c.381-40A > G (rs148673267) (0.0125), values close to those reported in population databases, with the exception of c.381-40A > G, which presented a higher AF in this study (Table 2).

In the *GATA4* gene, 27 variants, 6 reported in disease databases: 3 synonymous, 2 missense and 1 intronic (figure a). By means of in silico tools the 6 variants were classified as benign (Figure 2 Table 1).

The three variants with the highest AF were c.1132A > G (0.0406) (rs3729856), c.1000 + 23A > T (rs76808439) (0.0281), and c.1059C > T (rs3729855) (0.02188), with values close to those reported in population databases (Table 2).

In the *NOTCH1* gene, 219 variants, 82 reported in disease databases: 40 intronic, 28 synonymous, 13 missense and 1 UTR (Untranslated region) (figure a). By means of in silico tools 67 variants were classified as benign, 14 were reclassified from benign to VOUS (c.3171 + 42G > A, c.3325 + 26G > A, c.5167 + 189C > A, c.2604C > T, c.663C > T, c.7233A > G, c.3511-10G > A, c.5019-13A > G, c.2587 + 20G > A, c.2207 + 10G > A, c.6555C > T, c.3325 + 21A > G, c.*6G > A, c.5384 + 34G > A), and 1 was reclassified from benign to pathogenic (c.2354-24C > T, rs201516331) (figure b; Table 1) The three variants with the highest allelic frequency were recorded (Table 2)

In the *PTPN11* gene, 36 variants, 10 reported in disease databases: 9 intronic and 1 synonymous (figure a). By means of in silico tools, 7 benign variants were classified, 2 were reclassified from benign to VOUS (c.756 + 1274G > A, c.526-33_526-31del) and 1 was reclassified from benign to pathogenic (c.854-30T > C, rs144391508) (Figure 2; Table 2).

The three variants with the highest AF were c.854-30T > C (rs144391508) (0.0875), c.756 + 1274G > A (rs11066315) (0.0781) and c.854-21C > T (rs41279090) (0.028). the former obtained a higher AF in this study than in the population databases, the last two presented AF close to these databases (Table 2).

## 4 Discussion

Cellular events and genetic signaling are determining factors for cardiac development, since it is a complex process and any pathogenic variant in these genes can cause different CHD.

Variants in the *NKX2-5* gene represent 4 % of CHD causes [23]. The c.335-311_335-303del variant was reclassified by *in-silico* tools as probably pathogenic; however, it has not been previously reported in the literature. The c.63A > G variant has been related to atrial septal defect (ASD) in Indonesian patients with a frequency of 85.6 %, and in China with a frequency of 80.18 % [15,24], the c.335-311A > T and c.335-315A > T variants have not been previously reported in the literature.

For the variants with greater AF of *TBX20*, Santillán in 2015 [25], reports c.39T > C, c.545 + 13A > G and c.655-44G > A as gene variants associated with heart disease; c.655-44G > A was reported by Monroy et al. 2015 in patients with ASD [16]; c.381-40A > G has not been previously reported in the literature.

For the *GATA4* gene, the c.1132A > G variant was considered by Orjuela et al. [26] as “polymorphism”, however, Liu et al. [27] identified this variant in a heterozygous state in 2/600 patients in a Han Chinese population that presented a clinical diagnosis of cone truncal defects (CTD), one presented Tetralogy of Fallot and another pulmonary atresia with ventricular septal defect (VSD), without being described in the control population, also indicate that Al-Azzouny et al. [28] predicted that this variant could give rise to a non-functional transcript. Orjuela et al. [26], in a study of the Colombian population of 33 individuals with non-syndromic heart disease, reported this variant in a heterozygous state in two individuals, one with ASD and other with aortic coarctation, and Miuya et al. [29] report it present in patients with CHD. The c.1000 + 23A > T variant was found in a patient with tricuspid regurgitation and reported in a study of Moroccan patients with ASD, without presenting an effect on splicing [30,31]. The c.1059C > T variant was found in Moroccan patients with hypertension and acute myocardial infarction [29].

In the *NOTCH1* gene, the c.2354-24C > T variant was reclassified as pathogenic, and could theoretically have a pathogenic effect on splicing according to HSF, however, it has not yet been reported in the literature. the c.1669+9T > C variant was identified in patients clinically diagnosed with bicuspid aortic valve (BAV) [32]; Variants c.2970-31A > G and c.5473-43T > C have been found in patients with BAV/ascending aortic aneurysm [33,34]. BAV is one of the most common congenital anomalies. frequent affecting 1–2% of the general population; *NOTCH1* variants are implicated in aortic valve diseases [35].

In the *PTPN11* gene, the c.854-30T > C variant was reclassified as pathogenic; however, it has not been reported in the literature in association with CHD; this variant presented the highest AF; pathogenic variants do not necessarily imply disease, but rather a predisposition to segregation of the variant to future generations [36]. According to El Bouchikhi et al. [37], the c.854-21 C > T variant was indicated as recurrent in patients with Noonan syndrome, of whom 84 % presented CHD, mainly pulmonary valve stenosis. The c.756 + 1274G > A variant has not been previously reported in the literature.

Intronic variants were relevant in the TBX20, NOTCH1 and PTPN11 genes. This work contributes to knowledge about the frequency with which genomic variants associated with coronary heart disease are present in the population, which allows us to know those variants that are more frequent in the population and that could increase the risk of presenting congenital heart disease.

## 5 Conclusion

In this study, genomic variants associated with heart diseases were identified in the different genes involved in cardiac development in the population of southwestern Colombia in patients with complex disease and not clinically diagnosed with coronary heart disease. Reaching even the highest allele frequencies in genes such as NKX2-5, NOTCH1 and PTPN11, and some were reclassified as pathogenic according to in silico prediction tools, suggesting the need for further studies on the impact and frequency of these variants in the population with congenital heart disease to allow the reach of precision medicine.

## 6 Ethical statements

Protection of people and animals:

This work conforms to the principles set forth in the Declaration of Helsinki of the World Medical Association (WMA). The authors declare that this article does not involve direct participation with humans, due to the fact that a bioinformatic genomic analysis was performed on the results of complete exomes of patients from the southwest of Colombia after signing a consent form and informed consent, taken by the GENÓMICA Institute of Medical Genetics of the city of Cali.

## 7 Confidentiality data

The authors declare that no patient data appear in this article and have followed the protocols of their work center, receiving the corresponding informed consent in accordance with the protocol of the Genomics Medical Genetics Institute protocol of the Institute of Medical Genetics Genomics.

## 8 Limitations

This study only has a group of 320 exome sequencing results, which is sufficient but not totally representative of southwestern Colombia.

## Data availability statement

No - Data included in article/supp. material/referenced in article.

## CRediT authorship contribution statement

**Angie Lizeth Grueso Cerón:** Writing - review & editing, Writing - original draft, Methodology, Investigation, Formal analysis. **Daniela Arturo-Terranova:** Validation, Supervision, Project administration. **José María Satizábal Soto:** Validation, Supervision, Project administration, Conceptualization.

## Declaration of competing interest

The authors declare that they have no known competing financial interests or personal relationships that could have appeared to influence the work reported in this paper.
